# Risk Factors for Rotator Cuff Disease: An Experimental Study on Intact Human Subscapularis Tendons

**DOI:** 10.1002/jor.24385

**Published:** 2019-06-25

**Authors:** Fabian Plachel, Philipp Moroder, Renate Gehwolf, Herbert Tempfer, Andrea Wagner, Alexander Auffarth, Nicholas Matis, Stephan Pauly, Mark Tauber, Andreas Traweger

**Affiliations:** ^1^ Institute of Tendon and Bone Regeneration, Spinal Cord Injury & Tissue Regeneration Center Salzburg Paracelsus Medical University Salzburg Austria; ^2^ Department of Orthopedics and Traumatology Paracelsus Medical University Salzburg Austria; ^3^ Center for Musculoskeletal Surgery Charité Universitaetsmedizin Augustenburger Platz 1 Berlin 13353 Germany; ^4^ German Shoulder Centre ATOS Clinic Munich Munich Germany; ^5^ Austrian Cluster for Tissue Regeneration Vienna Austria

**Keywords:** rotator cuff tear, tendon degeneration, gene expression profiling, histological analysis, extrinsic risk factors

## Abstract

Although several studies revealed a multifactorial pathogenesis of degenerative rotator cuff disorders, the impact and interaction of extrinsic variables is still poorly understood. Thus, this study aimed at uncovering the effect of patient‐ and pathology‐specific risk factors that may contribute to degeneration of the rotator cuff tendons. Between 2015 and 2018, 54 patients who underwent arthroscopic shoulder surgery at three specialized shoulder clinics were prospectively included. Using tendon samples harvested from the macroscopically intact subscapularis (SSC) tendon, targeted messenger RNA expression profile analysis was performed in the first cohort (*n* = 38). Furthermore, histological analyses were conducted on tendon tissue samples obtained from a second cohort (*n* = 16). Overall, both study cohorts were comparable concerning patient demographics. Results were then analyzed with respect to specific extrinsic factors, such as patient age, body mass index, current as well as previous professions and sport activities, smoking habit, and systemic metabolic diseases. While patient age, sports‐activity level, and preexisting rotator cuff lesions were considered to contribute most strongly to tendinopathogenesis, no further coherences were found. With regards to gene expression analysis, change in expression correlated most strongly with patient age and severity of the rotator cuff pathology. Further, chronic disorders increased overall gene expression variation. Taken together, our study provides further evidence that tendon degeneration is the consequence of a multifactorial process and pathological changes of the supraspinatus tendon affect the quality of SSC tendon and most likely vice versa. Therefore, the rotator cuff tendons need to be considered as a unit when managing rotator cuff pathologies. © 2019 The Authors. *Journal of Orthopaedic Research* ^®^ published by Wiley Periodicals, Inc. on behalf of Orthopaedic Research Society J Orthop Res 38:182–191, 2020

High incidence rates and functional disability determine the overall significant socioeconomic impact of rotator cuff pathologies. Concerning the natural history of tendon disorders, rotator cuff tears (RCT) represent the final stage of a long‐lasting continuum.^1^ The vast majority of scientific studies revealed an atraumatic pathoetiology,^2^ related to degenerative changes within the tendon tissue secondary to patient‐specific characteristics, such as age and physical load.^3^, ^4^ Generally, increasing age has been shown to be a major contributing factor^5^ as the prevalence of RCTs rises markedly from the fourth decade of life onwards, affecting >50% in the elderly.^6^, ^7^ In addition, mechanical overuse is an additional major causative factor contributing to rotator cuff disorders.^8^ Recently, further anatomical (e.g., acromial shape^9^ or scapular dyskinesis) and environmental (e.g., obesity^10^ or metabolic syndrome^11^) characteristics have been indirectly associated with the onset of tendinopathy. Taken together, there is evidence to support the theory that initially mainly extrinsic variables contribute to and drive tendon degeneration. Subsequently, intrinsic processes concomitantly promote the pathological change of tendon tissue properties. On the basis of this multifactorial pathomechanism, early stages of rotator cuff lesions incorporate inflammatory changes and mislead healing processes.^12^ Although partially reversible, tendon disrepair due to repetitive microtrauma is followed by a loss of fiber organization, decreased or increased cellularity, calcification, lipid deposition, and cartilaginous or osseous metaplasia, which together characterize the irreversible final stage of degenerative tendinopathy, predisposing the tendon to rupture due to reduced tensile load capacities.^13^

While a large amount of experimental studies propagates distinct characteristic features of age‐related tendon degeneration, part of them might not be exclusively age‐specific. Therefore, analyzing the impact and interaction of extrinsic variables that may trigger rotator cuff degeneration is imperative for future efforts to developing diagnostic, preventive, and treatment strategies.

Current literature dealing with the pathoetiology of rotator cuff pathologies is mainly hampered by the fact that tendon tissues are usually studied in either animal models, human cadaver specimens, or surgical biopsies from degenerated or torn supraspinatus (SSP) tendons, limiting the significance of contributing pathogenic factors. Moreover, recent investigations revealed that a large number of cellular and molecular alterations are also found in control tendons, although macroscopically intact and clinically asymptomatic.^14^, ^15^ The aim of this study was therefore to assess the impact of patient‐ and pathology‐specific (extrinsic) risk factors on the degeneration of the macroscopically intact subscapularis (SSC) tendon, an imperative element for optimal shoulder function by ensuring dynamic glenohumeral stability. It was hypothized that SSC tendon degeneration is affected by several extrinsic characteristics, such as patient age, sex, smoking habit, body mass index (BMI), metabolic disease, physical activity, and glenohumeral injury as well as severity of additional rotator cuff pathologies.

## MATERIAL AND METHODS

Prior to the beginning of this prospective multicenter study (level of evidence: III), approval of the local ethical committee was obtained (EK‐2014‐1809). Each patient signed the written informed consent. Between 2015 and 2018, tissue samples from the intact SSC tendon were obtained from 54 patients who underwent arthroscopic shoulder surgery at the (i) the Department of Orthopedics and Traumatology (Paracelsus Medical University, Salzburg, Austria), (ii) Center for Musculoskeletal Surgery (Charité Universitaetsmedizin, Berlin, Germany), and (iii) German Shoulder Centre (ATOS Clinic, Munich, Germany). The arthroscopic procedure itself was indicated due to a preexisting pathology and not by the study protocol.

In general, men and women aged 18 or older with a clinically, radiologically, and arthroscopically intact SSC tendon at the time of surgery were included. Exclusion criteria were a structural lesion of the SSC tendon, a previous surgery on the affected shoulder, adhesive capsulitis, calcific tendonitis as well as symptomatic osteoarthritis of the affected shoulder. No further inclusion or exclusion criteria were applied.

### Clinical Characteristics

At time of initial presentation at the outpatient clinic, a standardized written questionnaire was completed to record patient‐specific information, such as (i) age at the time of surgery, (ii) gender, (iii) height and weight indicating the BMI, (iv) dominant as well as affected shoulder, (v) current as well as previous professions and sport activities (SAL score^16^), (vi) smoking habit (pack‐years), and (vii) systematic metabolic diseases (e.g., diabetes mellitus and thyroid dysfunction).

The SAL score was used to rate the individual shoulder‐specific exertion level. This cumulative rating scale consists of two items asking about (i) work‐ and (ii) sports‐related shoulder activity. The sum of the items (0–2 points each) is transformed into a score between 0 and 4 points, whereby 0 represents no shoulder‐specific activity and 4 extreme shoulder‐specific activity.^16^

Furthermore, a standardized clinical examination with subsequent analysis of magnetic resonance imaging (MRI) scans was performed to examine any soft‐tissue injuries of the affected shoulder joint and to rule out any clinically and radiologically detectable evidence of SSC tendon pathology.

### Tissue Collection

**Figure 1:**
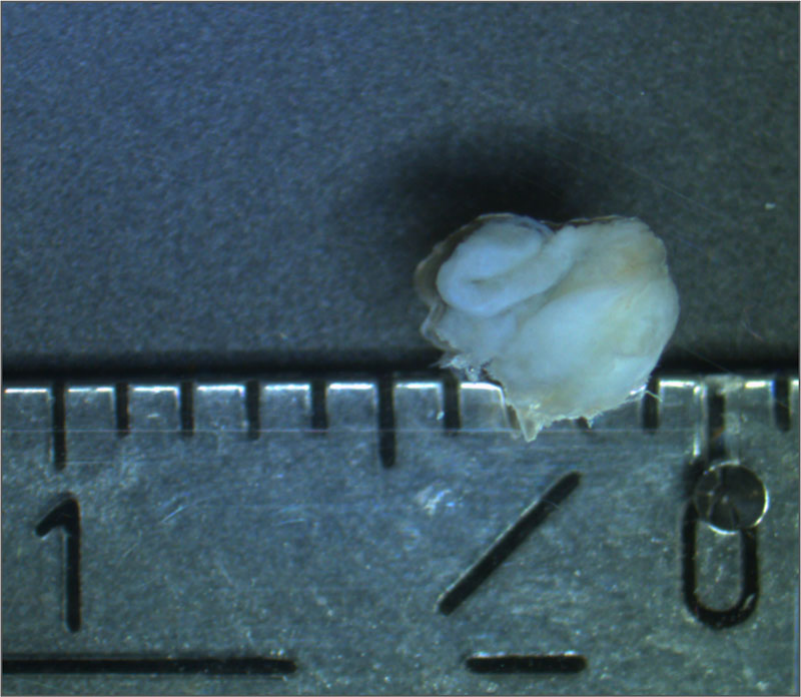
Image showing the biopsy obtained from the upper portion of the healthy subscapularis tendon with a dimension of approximately 2 × 2 × 2 mm. [Color figure can be viewed at wileyonlinelibrary.com].

Diagnostic arthroscopy was performed in a standard manner to evaluate the glenohumeral joint. The SSC tendon was critically assessed for any macroscopic damage. Tendon samples with a size of 2 × 2 × 2 mm (Fig. 1) were harvested from the upper portion of the intact SSC tendon, 10 mm lateral to the articular surface of the glenoid with the arm in neutral position. The site was photo documented prior to and after retrieving the biopsy. A custom‐made surgical punch was used. The biopsies were directly placed in a test tube containing (i) RNAlater (Qiagen, Hilden, Germany) for gene expression analysis (*n* = 38, first cohort) and (ii) 4% paraformaldehyde (PFA) for histological analysis (*n* = 16, second cohort), which were subsequently performed at the Institute of Tendon and Bone Regeneration (Paracelsus Medical University, Salzburg, Austria).

### Pathological Characteristics

As the study cohort (*n *= 54) was intentionally heterogenous in clinical as well as pathological characteristics, patients were incrementally separated into group 1 (pathology onset), defined as those suffering acute (1A; patients with an acute trauma resulting in any shoulder complaints; for example, traumatic glenohumeral dislocation) or chronic (1B; patients suffering from any non‐traumatic shoulder complaints for >12 months; for example, shoulder impingement syndrome) glenohumeral injuries,^17^ and group 2, defined by the severity of preexisting rotator cuff disorders; (2A) no rotator cuff (RC) disorder, (2B) partial posterosuperior (SSP tendon ± infraspinatus [ISP] tendon) RC disorder including subacromial impingement syndrome (not involving the SSC tendon), and (2C) full‐thickness posterosuperior RC tear (not involving the SSC tendon), respectively.

### RNA Extraction and Quantitative Polymerase Chain Reaction

First, total RNA was isolated from the tendon samples retrieved from the first cohort using TRI® Reagent (Sigma‐Aldrich, Vienna, Austria) according to the manufacturer's protocol, and RNA yield as well as purity were determined using a NanoDrop 2000c spectrophotometer (Thermo Fisher Scientific, Vienna, Austria). The quality of the RNA was assessed using an Experion RNA StdSens analysis kit (Bio‐Rad; Vienna, Austria). For first strand Complementary DNA (cDNA) synthesis, 1 µg total RNA was reversed transcribed using the iScript Reverse‐Transcription Supermix (Bio‐Rad). Subsequently, 5 ng of cDNA were analyzed by semi‐quantitative reverse‐transcription PCR (RT‐PCR) on a CFX 96 Touch Real Time Cycler (Bio‐Rad; Hercules, CA) using the TaqMan Gene Expression MasterMix chemistry (Life Technologies; Vienna, Austria). Amplification conditions were 50°C for 2 min, 95°C for 10 min, followed by 40 cycles of 95°C for 15 s, and 60°C for 1 min. All samples were run in duplicate and a minimum of two independent runs were performed.

On the basis of published data and unpublished preliminary results, a set of 16 genes was chosen for further analysis. Of those, COL1A1, COL3A1, matrix metallopeptidase 2 (MMP‐2), and LOX are related to extracellular matrix (ECM) composition and remodeling; MKX, SCX, and TNMD to tenogenic differentiation; COMP and SOX9 to chondrogenic differentiation; runt‐related transcription factor 2 (RUNX2) to osteoblastic differentiation; peroxisome proliferator‐activated receptor γ to adipocyte differentiation; and CD163, MRC1, TLR3, P2RX7, and TGFβ1 to inflammatory responses. Values were analyzed using qBasePlus 2.4 (Biogazelle NV, Zwijnaarde, Belgium) and normalized relative quantities were calculated by normalizing the data to the expression of four previously validated endogenous control genes (PUM1, RPLP0, TBP, and HPRT).

### Histology

For histological staining, the tissue samples retrieved from the second cohort were fixed in 4% PFA for a minimum of 12 h at 4°C and after extensive washing in phosphate‐buffered saline (PBS) and cryoprotection in 30% sucrose solution in PBS at 4°C samples were embedded in cryomedium (Tissue‐Tek O.C.T. Compound; Science Services, Munich, Germany). Subsequently, 12 µm cryosections were prepared (Leica CM1950; Leica, Vienna, Austria) and stored at −80°C until further use. For descriptive histology and assessment of the modified Bonar score,^18^ sections were stained either using hematoxylin and eosin or Alcian Blue stain. Using polarization microscopy, the alignment and organization of the collagen fibrils were investigated in order to objectively assess microstructural integrity. The same sections were scored by two examiners on two different days. In the case of disagreement, consensus was found after re‐analysis by using the means of both raters.

### Statistics

Descriptive statistics including means, standard deviations (SD), and minimum as well as maximum data of continuous variables were calculated. All data were assessed for normal distribution using the Kolmogorov–Smirnov test. Quantitative clinical characteristics, gene expression data, and the modified Bonar scores were compared between subgroups by either the independent *T* test for normally distributed data or the Wilcoxon test for non‐normally distributed data. Differences between more than two subgroups were assessed using the analysis of variance test. The relationships between patient‐ and pathology‐specific data and relative gene expression levels were estimated using either the Spearman's or Pearson's correlation coefficients. The *χ* ^2^ test was carried out to compare the distribution of nominal variables, such as sex and metabolic disease, between study cohorts or subgroups. Statistical analyses were performed using SPSS Statistics 21.0 (IBM, Armonk, NY). Multivariate analysis (Principal Component Analysis [PCA]; Partial Least Squares (PLS) Regression) was then performed using either ClustVis (https://www.biit.cs.ut.ee/clustvis/) or an Excel programmed data sheet (XLStat 2017; Addinsoft, Long Island City NY). For the analysis, the relative expression data for the stable endogenous controls were excluded. Graphs were plotted using either Microsoft Excel or GraphPad Prism. All tests were two‐sided, and *p *< 0.05 were considered statistically significant.

## RESULTS

### Patient Demographics

A total of 38 patients were enrolled for gene expression analysis between 2015 and 2017. The mean age at time of surgery was 50.6 ±  16.9 years (range, from 18 to 81 years). There were 30 male (79%) and 8 female (21%) patients. The mean BMI was 27.0 ± 4.0 (range, from 19.3 to 36.8). Overall, 15 patients (39%) had a positive smoking anamnesis with an average of 5.7 ± 10.7 pack‐years (range, from 1 to 52). A total of eight patients (21%) had a positive medical history for a systemic metabolic disease. The mean SAL at the time of surgery was 1.2 ± 1.0 points with a range from 0 to 3 points. Distributions in patient demographics between the pathology‐specific subgroups are summarized in Table 1.

**Table 1:** Patients Characteristics of Cohort 1 Related to the Subgroups

Variables	Group 1A (*N *= 8)	Group 1B (*N* = 30)	*p* Value	Group 2A (*N* = 13)	Group 2B (*N* = 13)	Group 2C (*N* = 12)	*p* Value
Patient age (years; mean ± SD)	34.0 ± 12.5	55.0 ± 15.3	0.001	32.8 ± 10.2	54.7 ± 12.7	65.4 ± 6.3	0.001
Body mass index (kg/m^2^; mean ± SD)	25.6 ± 3.0	27.3 ± 4.2	0.303	25.4 ± 3.9	27.9 ± 3.8	27.6 ± 4.2	0.219
Male gender (%)	100	73	0.100	92	69	75	0.376
Pack‐years (mean ± SD)	7.0 ± 11.6	5.3 ± 10.7	0.696	4.3 ± 9.5	10.0 ± 14.5	2.4 ± 5.0	0.183
Metabolic disease (%)	0	27	0.100	0	23	42	0.028
Sports activity level (mean ± SD)	1.4 ± 1.1	1.2 ± 0.9	0.594	1.3 ± 0.9	1.3 ± 0.9	1.0 ± 1.0	0.670

Group 1A, acute onset; Group 1B, chronic onset; Group 2A, no rotator cuff lesion; Group 2B, partial posterosuperior rotator cuff lesion; Group 2C, full‐thickness posterosuperior rotator cuff tear.

In addition, a total of 16 patients (14 male, 2 female) were included for descriptive histology in 2018. The mean age of the patients at the time of surgery was 40.7 ± 13.4 years (range, from 20 to 58 years) and the mean BMI was 26.1 ± 4.4 (range, from 19.4 to 35.2). Of whom, seven patients (44%) had a positive smoking anamnesis with an average of 4.6 ± 6.9 pack‐years (range, from 5 to 7) and 6% of the patients suffered a systematic metabolic disease. Regarding overhead sports activity and profession, the mean SAL was 1.3 ± 1.2 points (range, from 0 to 4 points). Further distributions among the subgroups are displayed in Table 2.

**Table 2:** Patients Characteristics of Cohort 2 Related to the Subgroups

Variables	Group 1A (*N* = 5)	Group 1B (N = 11)	*p* Value	Group 2A (*N* = 8)	Group 2B (*N* = 8)	Group 2C (*N* = 4)	*p* Value
Patient age (years; mean ± SD)	29.9 ± 7.4	45.6 ± 12.8	0.024	31.2 ± 11.2	45.4 ± 7.1	55.0 ± 3.7	0.003
Body mass index (kg/m^2^; mean ± SD)	25.4 ± 3.8	26.4 ± 4.9	0.700	24.7 ± 3.6	28.8 ± 4.0	26.3 ± 6.0	0.326
Male gender (%)	100	81	0.308	100	100	50	0.032
Pack‐years (mean ± SD)	0.0 ± 0.0	6.6 ± 7.4	0.014	2.5 ± 7.1	8.8 ± 8.5	4.5 ± 3.3	0.354
Metabolic disease (%)	0	9	0.486	0	33	0	0.202
Sports activity level (mean ± SD)	2.0 ± 1.2	0.9 ± 1.1	0.104	1.9 ± 1.2	1.0 ± 1.2	0.3 ± 0.5	0.081

Group 1A, acute onset; Group 1B, chronic onset; Group 2A, no rotator cuff lesion; Group 2B, partial posterosuperior rotator cuff lesion; Group 2C, full‐thickness posterosuperior rotator cuff tear.

Overall, the two study cohorts were comparable concerning patient demographics. While no significant difference was found for sex (*p *= 0.460), smoking habit (*p* = 0.708), BMI (*p* = 0.492), metabolic disease (*p* = 0.183), and physical activity (*p *= 0.937); only patient age deviated to some degree (mean ± SD: cohort 1, 50.6 ± 16.9 vs. cohort 2, 40.7 ± 13.4; *p *= 0.043).

### Gene Expression Analysis

Overall, a significant correlation was found between patient age and the relative expression of the metalloproteinase MMP‐2 (*R *= 0.330, *p *= 0.043) as well as the osteoblast transcription factor RUNX2 (*R *= 0.421, *p* = 0.009). Furthermore, the relative expression of both MMP‐2 (*R* = −0.326, *p *= 0.044) and the chondrocyte transcription factor SOX9 (*R* = −0.402, *p *= 0.029) was negatively correlated with SAL. Other than that, no further significant correlations were found (Table 3).

**Table 3:** Correlations Between a Set of Patient Characteristics (Cohort 1) and Gene Expression

Genes	Patient Age (years)	*p* Value	SAL Score (points)	*p* Value	BMI (kg/m^2^)	*p* Value	Pack‐Years (number)	*p* Value
CD163	0.29	0.08	−0.01	0.56	−0.12	0.46	−0.19	0.25
COL1A1	0.28	0.11	−0.29	0.09	0.09	0.63	−0.15	0.39
COL3A1	0.09	0.61	−0.19	0.24	−0.06	0.73	−0.23	0.17
COMP	0.11	0.53	−0.15	0.39	−0.05	0.75	0.01	0.98
LOX	0.23	0.17	0.01	0.93	0.11	0.51	−0.09	0.61
MKX	0.19	0.28	−0.11	0.51	−0.12	0.50	0.05	0.76
MMP‐2	0.33	0.04	**−**0.33	0.04	0.01	0.56	−0.01	0.98
MRC1	−0.07	0.68	0.14	0.41	−0.13	0.44	−0.13	0.42
P2RX7	0.32	0.06	−0.03	0.87	0.01	0.99	−0.19	0.25
PPARγ	−0.16	0.34	0.04	0.82	0.04	0.81	−0.20	0.23
RUNX2	0.42	0.01	−0.10	0.55	0.12	0.48	−0.15	0.37
SCX	0.06	0.74	−0.21	0.21	−0.07	0.70	−0.10	0.56
SOX9	0.07	0.71	−0.40	0.03	0.14	0.46	−0.02	0.92
TGBβ1	0.13	0.45	−0.04	0.79	−0.19	0.27	−0.30	0.06
TLR3	0.19	0.27	0.01	0.99	−0.20	0.24	−0.25	0.13
TNMD	0.14	0.41	−0.04	0.84	0.03	0.84	−0.12	0.48

BMI, body mass index; SAL, sports‐activity level.

**Figure 2:**
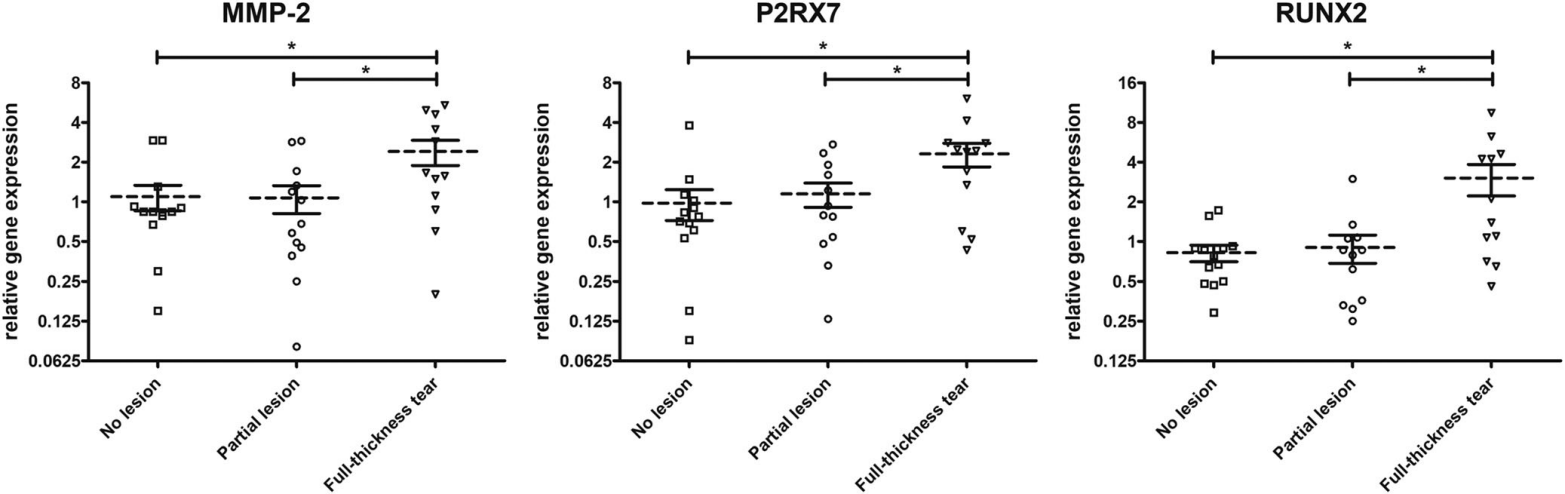
Scatterplots depicting the relative gene expression of three genes that were found to be differentially regulated with regards to the severity of preexisting posterosuperior rotator cuff lesions (*p < 0.05).

Interestingly, the relative gene expression data varied significantly across subgroups in both pathology‐related groups (Table 4). While the majority of genes was upregulated in tendon samples obtained from patients with chronic glenohumeral disorder (subgroup 1B) compared to those who suffered an acute injury (subgroup 1A), only the differences in relative expression values of the inflammatory‐related genes CD163 and P2RX7 as well as pro‐osteogenic RUNX2 were statistically significant (*p *< 0.05). Regarding subgroup 2, severity of tendon injury resulted in a significant upregulation of MMP‐2, P2RX7, and RUNX2 (*p* < 0.05) (Fig. 2). Overall, a steady increase in the relative expression levels toward RCT severity (subgroup 2) was found in the majority of the genes evaluated.

**Table 4:** Relative Gene Expression Values Correlated With the Subgroups

Gene Expression (Mean ± SD)	Group 1A	Group 1B	*p* Value	Group 2A	Group 2B	Group 2C	*p* Value
CD163	0.9 ± 0.4	1.5 ± 1.3	0.048	1.1 ± 0.9	1.1 ± 1.0	1.9 ± 1.5	0.139
COL1A1	1.1 ± 1.3	2.4 ± 3.3	0.339	1.1 ± 1.1	1.6 ± 2.7	3.7 ± 4.2	0.053
COL3A1	1.1 ± 0.9	1.7 ± 1.5	0.314	1.2 ± 0.8	1.4 ± 1.8	2.1 ± 1.5	0.246
COMP	1.6 ± 1.4	2.1 ± 3.3	0.652	1.4 ± 1.2	2.2 ± 3.5	2.5 ± 3.8	0.626
LOX	1.0 ± 0.8	1.9 ± 2.7	0.375	0.9 ± 0.7	1.8 ± 1.4	2.4 ± 4.0	0.312
MKX	1.2 ± 0.9	1.6 ± 1.8	0.577	1.2 ± 0.9	1.7 ± 1.5	1.8 ± 2.4	0.780
MMP‐2	1.2 ± 0.7	1.6 ± 1.5	0.256	1.1 ± 0.9	1.1 ± 0.9	2.4 ± 1.8	0.017
MRC1	0.8 ± 0.4	1.7 ± 1.9	0.176	1.4 ± 2.5	1.3 ± 0.6	1.8 ± 1.6	0.744
P2RX7	0.8 ± 0.5	1.7 ± 1.4	0.005	1.0 ± 0.9	1.1 ± 0.9	2.3 ± 1.6	0.016
PPARγ	1.7 ± 1.7	1.2 ± 0.9	0.348	1.6 ± 1.5	1.1 ± 0.7	1.3 ± 1.0	0.438
RUNX2	0.7 ± 0.4	1.7 ± 2.1	0.018	0.8 ± 0.4	0.9 ± 0.7	3.0 ± 2.8	0.003
SCX	1.0 ± 1.0	2.1 ± 2.7	0.269	1.4 ± 1.5	2.1 ± 3.2	2.3 ± 2.4	0.634
SOX9	0.9 ± 0.7	1.9 ± 3.4	0.443	0.9 ± 0.6	2.4 ± 4.8	1.6 ± 1.0	0.405
TGFβ‐1	0.9 ± 0.3	1.2 ± 0.7	0.064	1.0 ± 0.4	1.0 ± 0.8	1.5 ± 0.7	0.150
TLR3	1.1 ± 0.5	1.1 ± 0.8	0.964	1.0 ± 0.4	1.0 ± 0.7	1.4 ± 1.0	0.723
TNMD	2.4 ± 2.9	2.8 ± 3.1	0.729	2.1 ± 2.6	2.6 ± 3.0	3.5 ± 3.5	0.529

Group 1A, acute onset; Group 1B, chronic onset; Group 2A, no rotator cuff lesion; Group 2B, partial posterosuperior rotator cuff lesion; Group 2C, full‐thickness posterosuperior rotator cuff tear.

**Figure 3:**
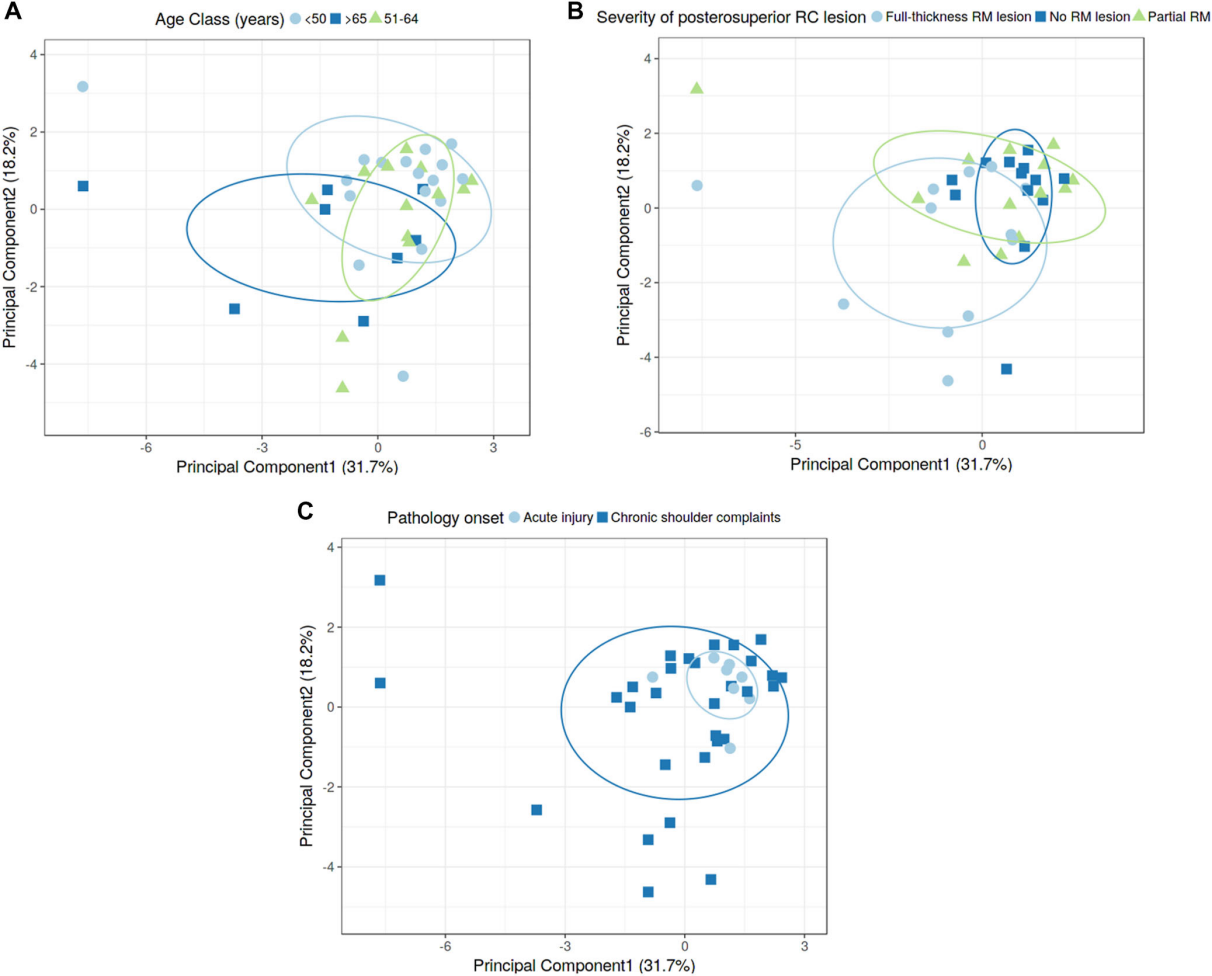
(A–C) Principal component analysis (PCA) of gene expression dataset. PCA biplot (score plot) visualizing projections onto the first two principal components (PC1 and PC2; circles indicate confidence interval of 50%). Each dot represents the gene expression of the 16 candidate genes determined for the SSC tissue samples: (A) patient age, (B) severity of preexisting posterosuperior rotator cuff lesion, (C) pathology onset. [Color figure can be viewed at wileyonlinelibrary.com].

Additionally, the dataset was interrogated using PCA to identify any type of structured variation contained in the dataset indicating an overall change of the gene expression pattern (i.e., 16 gene expression data per patient). The analysis revealed some degree of structured variation (49.9%), which was contained in the first two principal components (PCs). Although no absolute distinct clustering was evident, a moderate separation of the samples correlating with (i) age (Fig. 3A) and (ii) severity of rotator cuff disorder (Fig. 3B) was observed predominantly on PC1. Regarding glenohumeral pathology onset, PCA revealed a rather distinct expression pattern in patients with acute glenohumeral disorder, while chronic shoulder complaints resulted in a more heterogenous distribution (Fig. 3C).

As exploratory PCA analysis revealed structured variation within the dataset, we next sought to identify the gene expression patterns, which most strongly contributed to this variation using a PLS regression method. Genes with variable importance in projection scores greater than one were considered to contribute most strongly to the regression model based on the gene expression dataset. As shown in Table 5, indeed the relative gene expression of RUNX2, P2RX7, MMP‐2, MRC1, and COL1A1 are among the top three genes mainly contributing to the structured variation within the dataset with regards to the extrinsic factors (i) patient age, (ii) pathology onset, and (iii) severity of preexisting rotator cuff disorders.

**Table 5:** Genes Found to Strongly Contribute to the PLS Regression Model

Extrinsic Factor	Genes	VIP (Mean ± SD)
Patient age	RUNX2	1.891 ± 0.508
	MMP‐2	1.505 ± 0.219
	P2RX7	1.514 ± 0.471
	CD163	1.299 ± 0.629
	COL1A1	1.305 ± 0.540
	LOX	1.032 ± 0.683
Pathology onset	RUNX2	1.334 ± 0.771
	MRC1	1.331 ± 0.317
	P2RX7	1.733 ± 0.368
	CD163	1.162 ± 0.604
	COL1A1	1.015 ± 0.446
	SCX	1.089 ± 0.511
	TGFβ1	1.150 ± 0.474
Severity of preexisting rotator cuff disorders	RUNX2	1.465 ± 0.458
	MMP‐2	1.185 ± 0.378
	P2RX7	1.405 ± 0.458
	COL3A1	1.099 ± 0.337
	COL1A1	1.277 ± 0.261
	LOX	1.162 ± 0.666
	TGF‐β1	1.015 ± 0.531

PLS, partial least squares; SD, standard deviation; VIP, variable importance in projection.

### Histological Analysis

**Figure 4:**
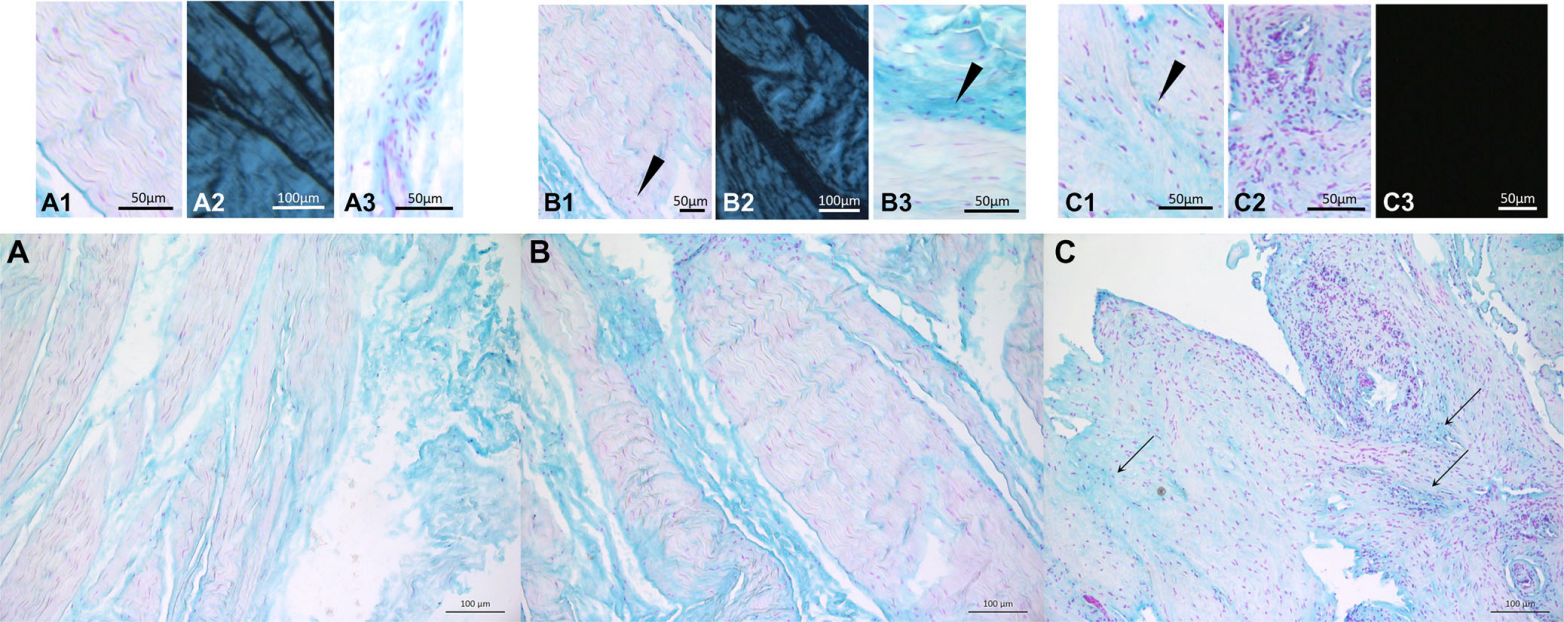
(A–C) Histological grading of tendon samples obtained from the intact subscapularis tendon. Scale bars are indicated. (A) No signs of tendinopathy (modified Bonar score: 0 points) were observed in a biopsy retrieved from a 29‐year‐old male patient without any signs of posterosuperior rotator cuff lesion (subgroup 2A). Bonar score characteristics: Cells with elongated cell nuclei, spindle shaped with no obvious cytoplasm (A1). Collagen arranged in tightly cohesive well demarcated bundles, with normal crimping (A2), little ground substance (blue stain), and inconspicuous presence of blood vessels (A3). (B) Moderate tendinopathy (modified Bonar score: seven points) in a 50‐year‐old male patient with a partial‐thickness lesion of the supraspinatus tendon (subgroup 2B). Bonar score characteristics: Hypocellularity (B1) and cells with round nuclei (arrowhead in B1), non‐homogeneous polarization pattern and diminished birefringence (B2), increased presence in ground substance (arrowhead in B3). (C) Severe tendinopathy (modified Bonar score: 16 points) was obvious in a 50‐year‐old male patient with a chronic full‐thickness tear of the supraspinatus tendon (subgroup 2C). Bonar score characteristics: Round nuclei with chondroid change (arrowhead in C1), mucin staining throughout the section (arrows in C), hypercellularization and increased vascularization (C2), and complete loss of birefringence (C3). [Color figure can be viewed at wileyonlinelibrary.com].

The modified Bonar score was assessed in intact SSC tendon samples (Table 6). The mean score was 7.2 ± 4.5 points (range, from 0.0 to 16.0 points). Overall, a significant correlation was found with patient age (*R* = 0.687, *p* = 0.005). Furthermore, SAL was negatively correlated with the modified Bonar score (*R* = −0.619, *p *= 0.010). No further patient‐specific characteristics correlated with the morphological degree of tendon degeneration (metabolic disease: *p* = 0.313; BMI: *p* = 0.436; smoking habit: *p *= 0.267). With regards to pathology‐specific data, there was a significant difference within the subgroup 1 (acute onset: 3.2 ± 2.1 points vs. chronic onset: 9.1 ± 4.0 points; 0.009) and subgroup 2 (no RC lesion: 3.7 ± 2.0 points vs. partial RC lesion: 9.7 ± 2.7 points vs. full‐thickness RC lesion: 11.9 ± 3.7 points; *p *< 0.001; see Fig. 4 and Table 6).

**Table 6:** Histological Scoring and Various Patient Characteristics of Cohort 2

Patient	Bonar Score	Patient Age	SAL Score	Subgroup 1	Subgroup 2
(number)	(points)	(years)	(points)	(1A vs. 1B)	(2A vs. 2B vs. 2C)
1	3	27	2	1A	2A
2	9	54	0	1B	2C
3	0	29	4	1A	2A
4	2	26	2	1B	2A
5	2	25	2	1A	2A
6	12	40	2	1B	2B
7	16	50	0	1B	2C
8	9	39	0	1B	2B
9	14	57	1	1B	2C
10	12	53	0	1B	2B
11	9	59	0	1B	2C
12	6	26	1	1A	2A
13	7	50	2	1B	2B
14	5	20	3	1B	2A
15	4	43	1	1A	2A
16	6	54	0	1B	2A

1A, acute glenohumeral injury; 1B, chronic glenohumeral disorder; 2A, no rotator cuff lesion; 2B, partial rotator cuff disorder; 2C, full‐thickness rotator cuff tear; SAL, sports‐activity level.

## DISCUSSION

The natural history of degenerative rotator cuff pathologies is still a matter of debate. While both clinical and experimental studies support a relationship between extrinsic factors and tendon disorders of the glenohumeral joint,^3^ their impact on pathophysiological processes responsible for tendon degeneration has not yet been fully unraveled. Therefore, this study aimed at defining extrinsic factors that may contribute to dysregulating tendon health.

Degenerative processes of the rotator cuff have been primarily investigated in the SSP tendon.^19^ However, as the majority of these studies have been conducted on ruptured SSP tendon, the change in gene expression profiles is most likely skewed due to intrinsic changes as a consequence of tendon rupture, rather than tendon degeneration itself.^3^, ^4^ In contrast, the SSC tendon is anatomically and mechanically interlinked to the SSP tendon and functional differences regarding strain, load, or inflammation within the shoulder joint also alter structural tendon characteristics and gene expression profiles in SSC tendons without concomitant SSC tissue damages.^12^, ^15^ Further, macroscopic discontinuities of the upper part of the SSC tendon have been reported,^20^ warranting further studies on SSC tendon degeneration. To the best of our knowledge, this is the first investigation using intact SSC tendon tissue samples from patients with varying degrees of shoulder disorder aiming at correlating patient‐ and pathology‐specific characteristics with molecular and cellular changes in tendon tissue. Based on our findings, patient age and the pathologic joint environment were found to contribute to SSC tendon degeneration.

For gene expression studies, we chose to investigate a set of 16 genes known to be involved in tendon homeostasis and degeneration. Next to investigating the change in expression of the genes individually, we also applied PCA. PCA is a statistical technique aiming at reducing the dimensionality of the data while simultaneously retaining the maximum amount of variance.^21^ Dimension reduction is achieved by calculating a set of new variables based on all possible pairwise interactions or interactions of higher order, which are termed PCs. PCs can reveal patterns in the original data, which might be overlooked if multivariate techniques are not applied. As shown in Figure 3, around 50% of the data variation was contained in the first two PCs. Although absolute distinct clustering was not evident, gene expression variability did increase with age (Fig. 3A), as well as severity of the RC lesion (Fig. 3B), which was mainly evident on PC1 (*x* axis). Similarly, PCA did reveal that the degree of expression variation was overall greater for tendon samples harvested from patients who had chronic shoulder pathologies, indicating the expression of the selected genes is more deregulated or heterogeneous in tendinopathic SSC tendon tissue. To identify those genes most strongly contributing to the data variability we performed partial least squares^22^ analysis, indicating that changes in expression of RUNX2, MMP‐2, COL1A1, and P2RX7 correlated most strongly with patient age and the severity of the rotator cuff disorders (i.e., no RC disorder; partial RC disorder including subacromial impingement syndrome; full‐thickness RC tear). Variation in gene expression for samples harvested from acute versus chronic RC‐diseased tendons most strongly correlated with P2RX7, MRC1, and RUNX2. P2RX7 is a ligand‐gated ionotropic receptor activated by extracellular adenosine triphosphate and has been shown to have an important role in multiple inflammatory and immune responses.^23^ MRC1 is required for clearance of mannose‐bearing serum glycoproteins that are normally elevated during inflammation.^24^ The expression of MRC1 is generally related to M2‐polarized macrophages, which are known to be important for the resolution of inflammation and the repair of damaged tissues. Taken together, although the change in expression of the individual genes was rather moderate, a chronic onset of RC pathology correlated with the elevated expression of P2RX7 and MRC1*,* both known to be involved in inflammatory processes, whereas patient age and severity of the RC pathology (e.g., subacromial impingement syndrome vs. full‐thickness RC tear) also correlated with genes encoding ECM and ECM‐remodeling proteins. Interestingly, for all patient groups investigated, an increase in the expression of RUNX2 was evident.

As also been shown by others,^5^ the results of our study demonstrated a clear association of patient age with a gradually increased severity of tendinopathy. Next to an increase in histological signs of tendon degeneration as evidenced by Bonar score analysis, also the relative expression of MMP‐2 and RUNX2 showed a significant age dependency. The expression of RUNX2 is directly linked to osseous metaplasia, a well‐known morphologic characteristic of severe tendon degeneration.^25^ In addition, multivariate data analysis revealed MMP‐2 expression to correlate with patient age (Table 5). MMPs have been extensively studied in the context of tendon pathologies^26^ as they are able to degrade the main ECM molecules in tendons, such as collagen type 1 and 3 and thus, regulate ECM turnover during tendon healing and remodeling. Healthy and diseased tendon tissues showed increased expression of several family enzymes, particularly MMP‐2, MMP‐9, and MMP‐19.^27^, ^28^ This is in accordance to our finding, further underscoring the key regulatory role of MMPs in connective tissue adaption.

Another well‐known extrinsic factor associated with tendon degeneration is mechanical overload.^29^, ^30^ For cohort 2 we found a moderately positive effect of physical activities on tendon health (Table 2). Interestingly, for those patients with the highest SAL score (3–4 points), only minor signs of tendon degeneration were seen by histological analysis (Bonar score: 0–5 points; Table 6). This might be due to the fact that the biomechanical function of the SSC tendon varies significantly from that of the SSP tendon. Furthermore, the SSC tendon is not regularly decompressed during active range of motion, preventing the tissue from repetitive microtrauma.^31^ Along these lines, Reeves et al. demonstrated that inactivity with further unloading had a negative effect on tendon collagen homeostasis and mechanical properties.^32^ Although patient numbers are small, our data would support the notion that overload might be a secondary contributing factor for tendon tears by propagating microinjuries or prior degenerative changes rather than being the main cause.^13^ However, as the negative correlation between SAL and RC severity was only seen in the second cohort by trend, a larger number of patients needs to be investigated to underscore this finding. Interestingly, a statistically significant negative correlation was also found between patient age and SAL (*R* = −0.316, *p *= 0.022; Spearman's rank‐order correlation). Therefore, age‐related processes in combination with less shoulder activity could together drive tendinopathic changes.

Although the SSC tendons included in our study appeared macroscopically normal, the proximity to the diseased rotator cuff seem to have provoked tendinopathic signs in the SSC tendon. Current studies revealed that full‐thickness rather than partial‐thickness SSP tendon ruptures were associated with synovial inflammation and tissue degeneration, suggesting that the joint is affected in its entirety rather than the tendon alone.^33^ Furthermore, Gausden et al.^34^ recently demonstrated an abnormal load transmission through the upper third of the SSC in the presence of concomitant RC disorder. Another investigation highlighted the impact of mechanical stress on early SSC tendinopathy, related to an inflammatory response that drive the tendon toward a degenerative process with active tissue remodeling.^14^ Consistently, relative mRNA levels for the majority of the genes investigated were found to be moderately higher in the group of patients who had suffered a full RC tear. Interestingly, while inflammatory genes were predominantly upregulated in the setting of full‐thickness SSP tendon ruptures, the expression of genes related to tenogenic differentiation (e.g., SCX and TNMD) was already elevated in response to “early stage” partial injuries. It was previously demonstrated that SCX stimulates TNMD as well as COL1A1 expression, which might be attributed to active ECM remodeling.^35^ A similar trend was found for the transcription factor TGFβ‐1,^36^ confirming the role in ECM remodeling by upregulating COL1A1 synthesis. Although COL1A1 is the major component of tendon ECM,^37^ increased messenger RNA levels for type 1 and 3 collagens were also found in diseased tendons.^38^

While recent studies propose an association between obesity, smoking habit, metabolic‐hormonal imbalances, and tendon degeneration,^10^, ^39^ our data do not support these findings. Therefore, most likely a combination of various demographic factors, rather than a single factor, underlie the development and progression of tendinopathy. However, we cannot exclude that those factors coincidentally impair tissue regeneration in a previously injured or degenerated tendon.

Our study has some limitations that need to be taken into consideration. First, the sample size is rather small. Nevertheless, to our knowledge, it is one of the largest cohort studies analyzing intact SSC tendon tissues of the glenohumeral joint. Furthermore, we were not able to simultaneously assess one tissue sample using gene expression analysis and histological analysis to directly compare the gene expression pattern with the stage of tendinopathy. This was primarily because biopsy samples were considered too small to split, as RNA quantity is also limited due to the relatively acellular nature of tendons. Nevertheless, our study demonstrates the feasibility to identify distinct genes expression patterns in biopsies harvested from the intact SSC. However, most likely a larger panel of genes needs to be interrogated to establish a more robust model. By defining a pattern based on these samples, gene expression analysis may be a useful tool for understanding the etiology and pathology of RCT and potentially to predict a future tear progression or a risk of failure of rotator cuff healing. Taken together, our findings are of great value to further increase our understanding of degenerative rotator cuff disorders.

## CONCLUSION

The results of our study further strengthen the notion that tendon degeneration is the consequence of a multifactorial process. Chronological age is most likely not the essential factor that needs to be considered for the progression of degenerative rotator cuff disorders. RC tendinopathy is rather driven by a combination of tissue senescence/degeneration processes and altered mechanical stimuli, as a consequence of chronic glenohumeral injuries and less loading activity with increasing age. Further, our analysis clearly shows that inflammatory processes and altered mechanical properties in the SSP tendon affect the remainder of the rotator cuff tendons. Therefore, for treatment of RC pathologies the entire functional unit needs to be taken into consideration.

## AUTHORS’ CONTRIBUTION

All listed authors have contributed substantially to this work (FP, PM, and AT conceived the study; FP, RG, HT, AW, and AT designed the experiments; FP, PM, AA, NM, SP, and MT recruited patients and collected tendon samples; FP, RG, HT, AW, and AT conducted the experiments; FP, PM, RG, HT, AW, and AT analyzed the data; all authors contributed to manuscript preparation and critically revised the manuscript). All authors approved the final manuscript and the submission.

## ACKNOWLEDGMENTS

This work was supported by the European Society for Surgery of the Shoulder and the Elbow (ESSE/SECEC; Research Grant 2015), the Lorenz Böhler Fund (Vienna, Austria, project number 1.15), and the Fund for the Advancement of Scientific Research at Paracelsus Medical University (PMU‐FFF, Salzburg, Austria).
